# The Effect of Uncoated SPIONs on hiPSC-Differentiated Endothelial Cells

**DOI:** 10.3390/ijms20143536

**Published:** 2019-07-19

**Authors:** Barbara Salingova, Pavel Simara, Pavel Matula, Lenka Zajickova, Petr Synek, Ondrej Jasek, Lenka Veverkova, Miroslava Sedlackova, Zuzana Nichtova, Irena Koutna

**Affiliations:** 1 Centre for Biomedical Image Analysis, Faculty of Informatics, Masaryk University, Kamenice 5, 62500 Brno, Czech Republic; 2 RG Plasma Technologies, CEITEC—Masaryk University, Purkynova 656/123, 61200 Brno, Czech Republic; 3 Department of Physical Electronics, Faculty of Science, Masaryk University, Kotlarska 2, 60200 Brno, Czech Republic; 4 1st Surgical Department, Faculty of Medicine, Masaryk University and St. Ann’s Hospital, Pekarska 53, 65691 Brno, Czech Republic; 5 Department of Histology and Embryology, Faculty of Medicine, Masaryk University, Kamenice 5, 62500 Brno, Czech Republic; 6 Institute of Molecular Physiology and Genetics, Slovak Academy of Sciences, Dubravska Cesta 9, 84005 Bratislava, Slovak Republic

**Keywords:** superparamagnetic iron-oxide nanoparticles, human induced pluripotent stem cell-derived endothelial cells, reprogramming, differentiation, mature endothelial cells

## Abstract

Background: Endothelial progenitor cells (EPCs) were indicated in vascular repair, angiogenesis of ischemic organs, and inhibition of formation of initial hyperplasia. Differentiation of endothelial cells (ECs) from human induced pluripotent stem cells (hiPSC)-derived endothelial cells (hiPSC-ECs) provides an unlimited supply for clinical application. Furthermore, magnetic cell labelling offers an effective way of targeting and visualization of hiPSC-ECs and is the next step towards in vivo studies. Methods: ECs were differentiated from hiPSCs and labelled with uncoated superparamagnetic iron-oxide nanoparticles (uSPIONs). uSPION uptake was compared between hiPSC-ECs and mature ECs isolated from patients by software analysis of microscopy pictures after Prussian blue cell staining. The acute and long-term cytotoxic effects of uSPIONs were evaluated by MTT (3-(4,5-dimethylthiazol-2-yl)-2,5-diphenyltetrazolium bromide assay) and Annexin assay. Results: We showed, for the first time, uptake of uncoated SPIONs (uSPIONs) by hiPSC-ECs. In comparison with mature ECs of identical genetic background hiPSC-ECs showed lower uSPION uptake. However, all the studied endothelial cells were effectively labelled and showed magnetic properties even with low labelling concentration of uSPIONs. uSPIONs prepared by microwave plasma synthesis did not show any cytotoxicity nor impair endothelial properties. Conclusion: We show that hiPSC-ECs labelling with low concentration of uSPIONs is feasible and does not show any toxic effects in vitro, which is an important step towards animal studies.

## 1. Introduction

Endothelial cell therapies show great potential in treatment of multiple diseases. Endothelial progenitor cells (EPCs) were indicated in vascular repair, angiogenesis of ischemic organs, and inhibiting the formation of intimal hyperplasia [1,2]. First preclinical studies on animal subjects report positive effects on ischemic myocardium, limb neovascularization or peripheral vascular regeneration [3,4,5]. Clinical application of EPCs is however limited by the small number of cells present in circulating blood. This number is further decreased in some diseases [6,7]. To overcome this issue, pluripotent stem cells have been investigated as an alternative cell source for therapies [8,9,10]. Reprogramming and differentiation provides an unlimited supply of patient specific endothelial cells [9,11,12,13]. Protocols for differentiation of functional endothelial cells have already been established and human induced pluripotent stem cell-derived endothelial cells (hiPSC-ECs) were shown to line a tissue graft suitable for implantation [10,11,14,15]. The next step in the therapy-oriented research is optimization of cell labelling with magnetic nanoparticles for cell guiding and tracking in vivo. Cell labelling is a convenient tool for detailed study of endothelial cell function in vivo, and cell tracking and guiding mechanisms for regeneration therapy. Moreover, it can provide valuable information about membrane properties of hiPSC-ECs, which were not studied until now.

For cell tracking and guiding mechanisms cells are magnetically labelled in vitro, where superparamagnetic iron-oxide nanoparticles (SPIONs) are uptaken through endocytosis resulting in a cell population susceptible to magnetic field. This cell population can be visualized and manipulated in vivo based on magnetic properties [16]. SPIONs for biomedical applications are usually formed by an iron oxide core and an organic coating. Several products were approved by FDA (Food and Drug Administration) for clinical studies to this day including Endorem (Ferumoxides), Resovist (Ferucarbotran) and Feraheme (Ferumoxytol) [17,18,19,20]. All three products were previously studied and used for magnetic labelling of circulating EPC. Resovist and Endorem showed 100% labelling and magnetic properties detectable by MRI after 24 h of incubation at concentrations of 100 µg/mL and 300 µg/mL respectively [19]. No toxicity was detected even under much higher concentrations of the labelling agents. However, labelling concentrations for nanoparticles remain high and new mechanisms are considered for more effective labelling with better biocompatibility.

The cellular uptake of SPIONs depends on several physicochemical parameters including size, shape, polydispersity, charge and coating. Coating of SPIONs is considered highly advantageous as it offers the possibility to affect nanoparticle properties, however it substantially changes the size and magnetic properties of the nanoparticles [21,22]. In some cases an iron-oxide core of size around 10 nm can result in a nanoparticles ranging from 80 nm to 150 nm dependent on organic coating, which significantly affects the efficiency of uptake and magnetic properties of the labelled cell [22,23,24]. Therefore, uncoated nanoparticles could offer better efficiency of uptake as their size is not affected by coating. Moreover, while it was previously believed that uncoated SPIONs (uSPIONs) have higher toxicity compared to coated nanoparticles, later it was reported that incubation of nanoparticles in serum prevents surface reactivity and results in decreased cytotoxicity [25].

The aim of this study was to prove the feasibility of hiPSC-EC magnetic labeling. Furthermore, as a control, we labeled mature ECs isolated from patients, which represent a cell model most closely resembling endothelial cells in vivo. We assessed the differences in uSPIONs uptake between human dermal fibroblasts (hDFs), mature EC (human umbilical vein endothelial cells (HUVECs), human saphena vein endothelial cells (HSVECs)) and hiPSC-ECs. We further theorized that uSPION could have superior magnetic properties over coated SPIONs and lower concentrations of uSPIONs would be required for cell labelling. Therefore, we evaluated the effectivity of uSPION endocytosis under low uSPION concentrations and magnetic properties of the labelled cells. We extensively studied uSPION cytotoxicity including toxic effects during the incubation and long-term effects after the incubation. Finally, we studied the effects of uSPIONs on cell characteristics desired for clinical application; specifically, their endothelial profile and angiogenic properties.

## 2. Results

### 2.1. Uptake of uSPIONs Is Different between Cell Types

**Figure 1:**
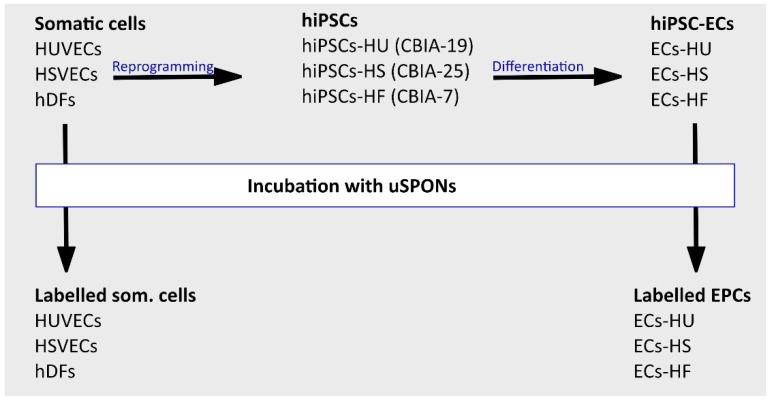
Experimental design. Three somatic cell lines were reprogrammed to pluripotent state (human induced pluripotent stem cells; hiPSCs) and differentiated into hiPSC-derived endothelial cells (hiPSC-ECs). Somatic cells and hiPSC-ECs were labeled with uncoated superparamagnetic iron-oxide nanoparticles (uSPIONs) and compared. Abbreviations: HUVECs, human umbilical vein endothelial cells; HSVECs, human saphenous vein endothelial cells; hDFs, adult human dermal fibroblasts; hiPSCs-HU, induced pluripotent stem cells derived from HUVECs; hiPSCs-HS, induced pluripotent stem cells derived from HSVECs; hiPSCs-HF, induced pluripotent stem cells derived from hDFs; ECs-HU, endothelial cells differentiated from hiPSCs-HU; ECs-HS, endothelial cells differentiated from hiPSCs-HS; ECs-HF, endothelial cells differentiated from hiPSCs-HF.

**Figure 2:**
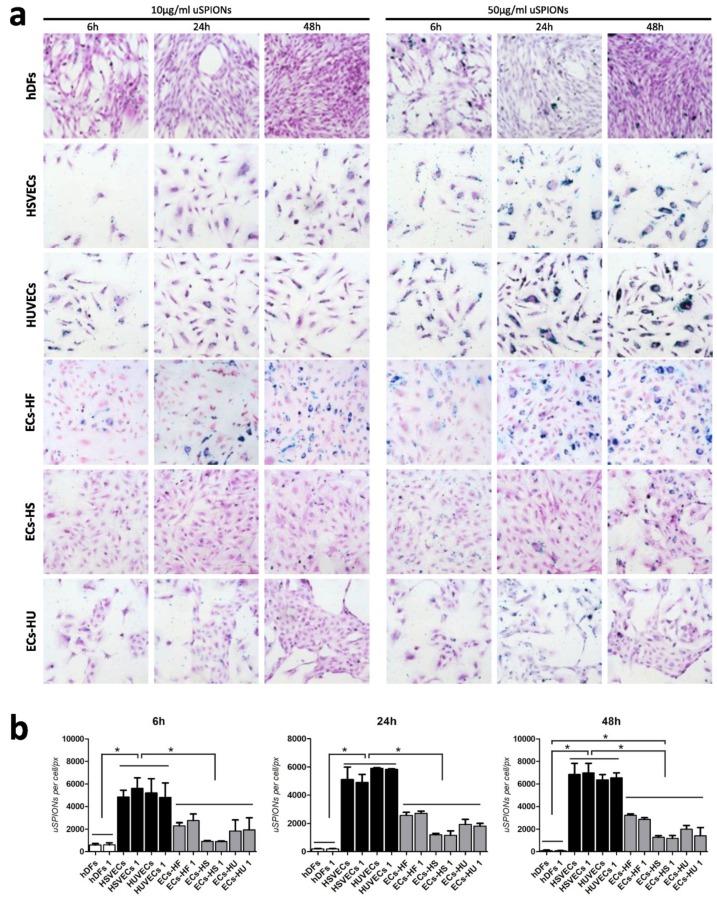
Internalization of uSPIONs in six different cell types. (**a**) Representative pictures of internalization of uSPIONs visualized after Prussian blue staining. Cells were incubated with uSPIONs for 6 h, 24 h and 48 h, stained with Prussian blue for iron detection and observed under a light microscope. Differences in the effectiveness of uSPION uptake can be seen between HUVECs/HSVECs and hiPSC-ECs—ECs-HU, ECs-HS, ECs-HF. No uptake was observed in hDFs. (**b**) Image analysis of internalization of uSPIONs in six different cell types and their biological replicates. Cells were incubated with 50 µg/mL of uSPIONs for 6 h (left), 24 h (right) and 48 h (bottom) (*n* = 3 ± SEM). No significant difference was observed between the two mature endothelial cell lines—HUVECs (1) and HSVECs (1)—or between three differentiated endothelial cells—ECs-HU (1), ECs-HS (1) and ECs-HF (1). A significant difference in uSPION uptake was shown between hDFs (1) and HSVECs (1)/HUVECs (1) and between HUVECs (1)/HSVECs (1) and ECs-HF (1)/ECs-HS (1)/ECs-HU (1) after 6, 24 and 48 h of incubation with uSPIONs (analyzed by one-way ANOVA followed by Tukey’s test, *p* > 0.05). Abbreviations: HUVECs, human umbilical vein endothelial cells; HSVECs, human saphenous vein endothelial cells; hDFs, adult human dermal fibroblasts; ECs-HUs, endothelial cells differentiated from hiPSCs-HU; ECs-HS, endothelial cells differentiated from hiPSCs-HS; ECs-HF, endothelial cells differentiated from hiPSCs-HF.

We reprogrammed HUVECs, HSVECs and hDFs into hiPSCs and differentiated them into hiPSC-ECs. We incubated six cell types (HUVECs, HSVECs, hDFs, ECs-HU, ECs-HS, ECs-HF) with 10 µg/mL and 50 µg/mL of uSPIONs. The experimental design is summarized in Figure 1. We repeated the process with biological replicates HUVECs 1, HSVECs 1, hDFs 1 to ensure reproducibility. We observed the intracellular uptake of uSPIONs after Prussian blue staining (Figure 2a). We showed different uptakes of uSPIONs in the studied cell types. In general, the quantity of uSPIONs uptaken by the cell increased with time and concentration of uSPIONs in medium. The first uptake was detected after 6 h of incubation. We did not observe any point of saturation that would render cells unable to take up more uSPIONs. 

We quantified the number of uSPIONs by custom software developed by our group (for details see methods and Figure S4). The differences in uSPION uptake were most apparent at higher concentrations of uSPIONs (50 µg/mL) (Figure 2b). The six cell lines and their replicates were divided into three groups according to uSPION uptake: hDFs/hDFs 1 did not show any uSPION uptake, mature ECs (HSVECs/HSVECs 1 and HUVECs/HUVECs 1) showed high uSPION uptake and hiPSC-ECs (differentiated from all three cell types) showed significantly lower uSPION uptake, relative to mature ECs. We did not observe any significant differences in uSPION uptake among the three hiPSC-EC lines. This suggests that the membrane properties of the original source cell type used for cell reprogramming do not have any effects on the properties of the differentiated ECs. We observed the difference in uptake of uSPIONs between ECs and hiPSCs-EC with identical genetic background. Reprogramming and differentiation changed the properties of cell membranes.

### 2.2. Biodistribution of the uSPIONs Observed by Transmission Electron Microscopy

**Figure 3:**
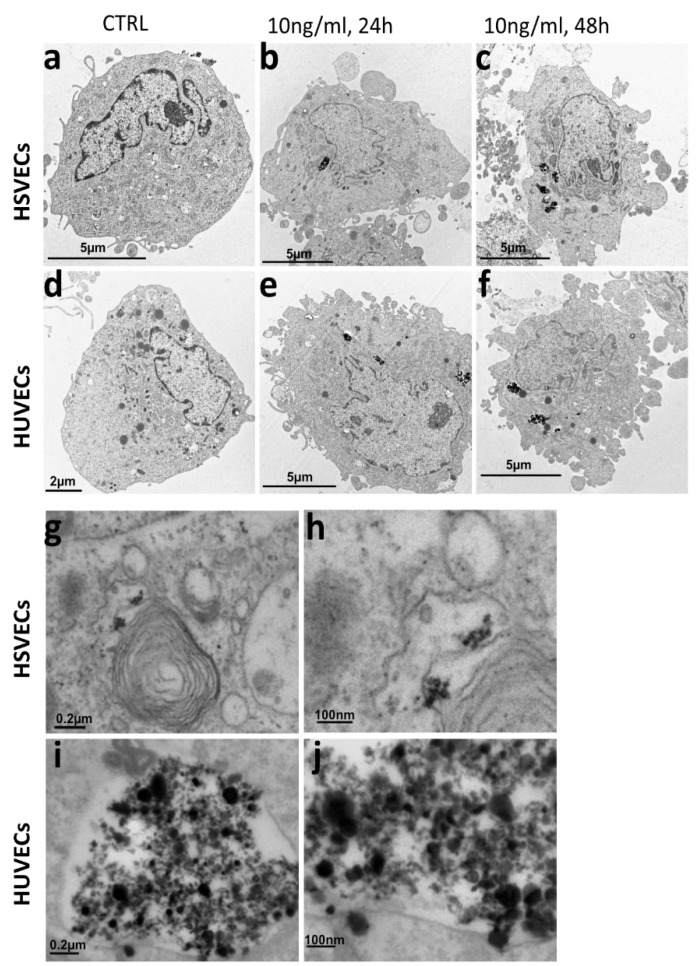
TEM of endothelial cells exposed to uSPIONs. Representative images of cells exposed to 10 ng/mL uSPIONs. (**a**) HUVECs control (without uSPIONs). (**b**) HUVECs incubated with 10 μg/mL uSPIONs for 24 h. (**c**) HUVECs incubated with 10 μg/mL uSPIONs for 48 h. (**d**) HSVECs control (without uSPIONs). (**e**) HSVECs incubated with 10 μg/mL uSPIONs for 24 h. (**f**) HSVECs incubated with 10 μg/mL uSPIONs for 48 h. (**g**,**h**) Details of internalized uSPIONs in vacuoles with myelin-like content. (**i**,**j**) Detail of internalized uSPIONs in vacuoles. uSPION size varies 20–100 nm. Abbreviations: HUVECs, human umbilical vein endothelial cells; HSVECs, human saphenous vein endothelial cells; hDFs, adult human dermal fibroblasts; ECs-HUs, endothelial cells differentiated from hiPSCs-HU; ECs-HS, endothelial cells differentiated from hiPSCs-HS; ECs-HF, endothelial cells differentiated from hiPSCs-HF.

Transmission electron microscopy (TEM) represents a definite confirmation of nanoparticle uptake and allows to assess uSPIONs size after uptake and their intracellular biodistribution. We incubated cells with 10 μg/mL uSPIONs for 24 h and 48 h, fixed the cells and visualized them by TEM. All the observed cell types were able to uptake uSPIONs (Figure 3a–f). 

The size of the uSPION was approximately 20 nm, and the variability in size was the result of aggregation (Figure 3i,j). uSPIONs were localized to cytoplasmic intracellular vesicles identified as autophagic vacuoles by their myelin-like content (Figure 3g,h). They entered the cell separately or in small aggregates and formed endocytic vesicles, which later fused together. The size of uSPIONs measured by Raman spectrometry was between 20–50 nm which corresponds with the size observed by TEM after cellular uptake (Figure S1).

### 2.3. ECs Show and Maintain Magnetic Properties after Labeling

**Figure 4:**
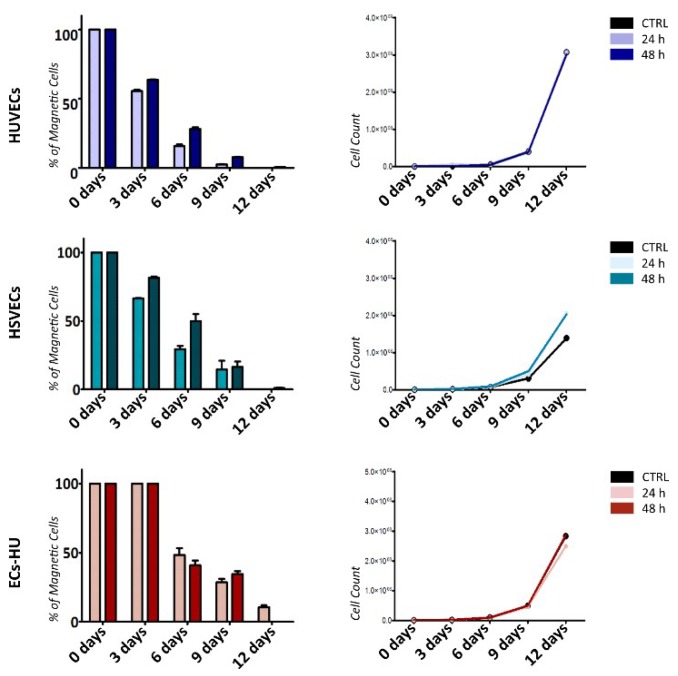
Cell magnetic properties of after incubation with uSPIONs. HUVECs, HSVECs and ECs-HU were incubated with 10 µg/mL of uSPIONs for 24 and 48 h. Graphs show quantity of magnetic cells (with the ability to be magnetically separated) expressed as a percentage of the entire cell population (left). *n* = 3 ± SEM. Expansion curve of the cell population (right). Abbreviations: HUVECs, human umbilical vein endothelial cells; HSVECs, human saphenous vein endothelial cells; ECs-HUs, endothelial cells differentiated from hiPSCs-HU.

We studied magnetic properties of the mature ECs (HUVECs/HSVECs) and hiPSC-ECs (EC-HU) immediately following the labeling and 3, 6, 9 and 12 days after labelling. We incubated HUVECs, HSVECs and ECs-HU 24 h or 48 h with 10 μg/mL of uSPIONs and separated them according to their magnetic properties by MACS separation. The data are shown as the percentage of magnetically separated cells from the cell culture. All HUVECs, HSVECs and ECs-HU were separated after 24 h and 48 h of incubation with uSPIONs (Figure 4). We measured the magnetic properties of the cell culture 3, 6, 9 and 12 days after incubation with uSPIONs. Cells retained their magnetic properties. The number of magnetic cells was proportional to the growth of the cell population. We were not able to observe decrease in number of magnetic cells. 

### 2.4. Exposure to uSPIONs does not Affect Cell Survival of ECs and hiPSC-ECs

**Figure 5:**
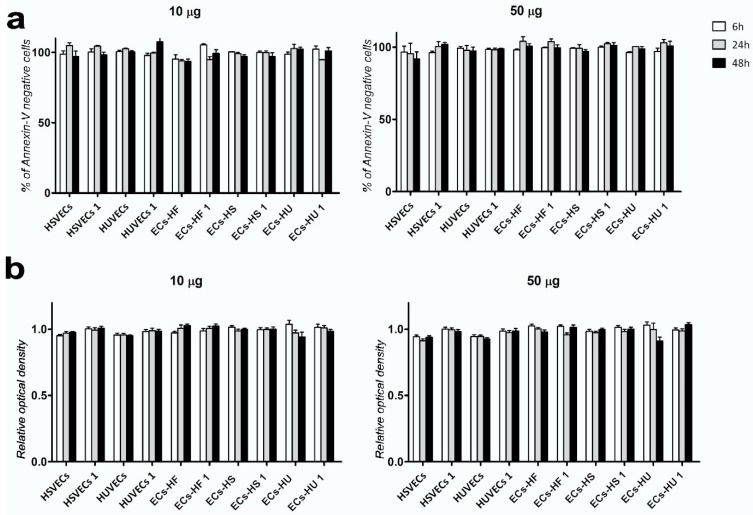
Cell viability assessed by Annexin (**a**) and MTT (**b**) assays. (**a**) Annexin assay following 6, 24 and 48 h of incubation with 10 µg/mL and 50 µg/mL of uSPIONs. Annexin A negative cells are expressed as percentage change in relation to control (cells that were not incubated with uSPIONs). (**b**) MTT assay following 6, 24 and 48 h of incubation with 10 µg/mL and 50 µg/mL of uSPIONs. Relative optical density is shown as a ration to control (CTRL = 1, cells that were not incubated with uSPIONs; *n* = 3 ± SEM). Statistical evaluation (*t*-test) showed no significant difference in viability among cell lines (*p* > 0.05) in any of the assays. Abbreviations: HUVECs, human umbilical vein endothelial cells; HSVECs, human saphenous vein endothelial cells; ECs-HUs, endothelial cells differentiated from hiPSCs-HU; ECs-HS, endothelial cells differentiated from hiPSCs-HS; ECs-HF, endothelial cells differentiated from hiPSCs-HF.

We assessed acute cytotoxicity and long-term cell survival during incubation with uSPIONs in ECs and hiPSC-ECs. MTT (3-(4,5-demethyltiazol-2-yl-2,5-diphenyltetrazolium bromide)) and Annexin assays were used to measure cell viability and metabolism. The MTT assay reflects the overall condition of the cell population through evaluation of cell metabolism. The Annexin assay specifically measures markers of early apoptosis and cell death. The cell cultures showed 90–100% viability in relation to the control sample in both assays (Figure 5). Cell viability was not significantly affected by magnetic labeling. Cells were also examined by light microscopy and did not show any morphological changes indicative of cell death. We studied the long-term effects of 24 and 48 h incubation with uSPIONs on the viability of the cell population. We did not observe any change in cell viability up to 12 days after incubation with uSPIONs (Figure S2). 

We also assessed cell survival during long-term continual exposure to uSPIONs (12 days) in cells that showed the highest uSPIONs uptake: HUVECs and HSVECs. Prolonged exposure to uSPIONs had no effect on cell survival (Figure S3).

### 2.5. Labelled ECs and EPCs Retain Endothelial Markers and Angiogenic Properties

**Figure 6:**
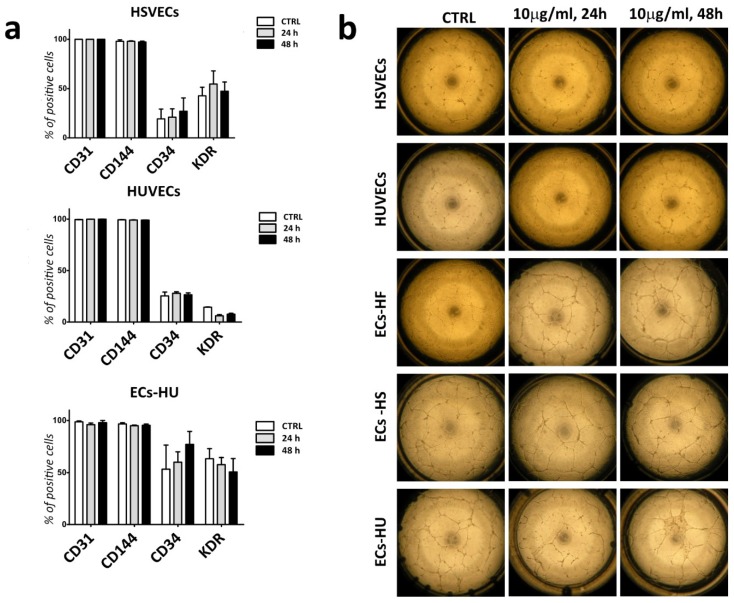
Flow-cytometry analysis of extracellular markers and tube formation assay after incubation with uSPIONs (**a**) Flow-cytometry analysis of extracellular markers of HUVECs, HSVECs and ECs-HU after 24 and 48 h of incubation with uSPIONs. CTRL corresponds to cell population that was not incubated with uSPIONs. (*n* = 3 ± SEM). Statistical evaluation (*t*-test) showed no significant change in expression of endothelial markers between cell lines (*p* > 0.05). (**b**) Tube formation assay following 24 and 48 h of incubation with 10 µg/mL of uSPIONs and control (cells without incubation with uSPIONs). Abbreviations: HUVECs, human umbilical vein endothelial cells; HSVECs, human saphenous vein endothelial cells; ECs-HUs, endothelial cells differentiated from hiPSCs-HU; ECs-HS, endothelial cells differentiated from hiPSCs-HS; ECs-HF, endothelial cells differentiated from hiPSCs-HF.

We studied the effect of uSPIONs on the phenotype and functionality of the cells. We analyzed the expression of endothelial characteristics markers in HUVECs, HSVECs and ECs-HU after 24 and 48 h of incubation with uSPIONs by FACS analysis. We did not observe any effect of uSPIONs on either the expression of CD31 and CD144 (markers confirming endothelial identity) or CD34 and KDR (markers indicative of unmature endothelial phenotype) (Figure 6a). We also evaluated angiogenic properties of the labelled cells by tube formation assay. We did not detect any effect of the uSPIONs on the angiogenic properties of either mature ECs or ECs-HU (Figure 6b). 

## 3. Discussion

SPIONs have become a useful tool for biomedical research and some are already in clinical use. They could provide an efficient system for cell tracking and guiding in therapy. However, labelling efficiency and the resulting magnetic properties of the labelled cells are of major concern. Currently used SPIONs show low uptake efficiency, which requires high concentration for labelling and the use of a transfection agent [19,26]. 

Coating significantly affects size of SPIONs as well as their magnetic properties [2,8,10]. We hypothesized that uSPIONs due to the absence of coating could possess superior magnetic properties and therefore lower concentration of nanoparticles would be needed for effective labelling. It was reported that uncoated SPIONs have low labelling efficiency in comparison with coated SPIONs [27]. However, we showed that a concentration as low as 10 µg/mL is sufficient to label and magnetically separate 100% of mature endothelial cells as well as hiPSC-ECs. In contrast, the reported labelling concentrations of coated SPION range from 20 µg/mL to 300 µg/mL [19,28].

We additionally pre-treated the uSPIONs with FBS (Fetal Bovine Serum). It was reported that coating reduces toxic effect of uSPIONs [25]. Pre-treatment with FBS causes formation of protein corona, that can affect the endocytosis of the nanoparticles, but protects the cell from toxic interactions with nanoparticle core [25,29,30,31]. 

In endothelial cells clathrin and caveolae-mediated endocytosis is the preferred way of cell entry [32]. It was repeatedly described that SPIONs in general use the same receptors for uptake [22,33,34]. Clathrin endocytosis occurs via a clathrin-assembly unit; nanoparticle binding initiates the formation of ‘coated pits’ on the cytoplasmic side of cell membrane. The pits later self-assemble to polygonal cages. Clathrin complex is also responsible for necking and pinch-off process [35,36]. Clathrin-mediated endocytosis produces vesicles of 100–150 [33]. In contrast caveolae-dependent endocytosis occurs in flask-shaped membrane invaginations lined with caveolin 50–80 nm in size [33]. The two major differences between caveolae and clathrin-mediated endocytosis are the size of the vesicles they form and the fact that clathrin-dependent endocytosis pathway often ends in lysosomal fusion and exocytosis.

We studied the rate of uSPION uptake under high and low concentration in hDFs, mature ECs and hiPSC-ECs. Mature ECs (HUVECs and HSVECs) showed the highest effectiveness in uSPION uptake, hiPSC-ECs showed significantly lower uSPION uptake than mature ECs and no uptake could be observed in hDFs. SPIONs uptake is dependent mainly on adsorption to cell membrane and clathrin or caveolae-mediated endocytosis [22,32,34]. This proposed mechanism can explain our observations while hDFs and ECs have significantly different membrane composition and ECs show an increased number of clathrin and caveolae receptors in comparison to hDFs. 

hiPSC-ECs were never studied in the past with respect to their membrane properties. We were interested in whether the original tissue used for reprogramming can affect the properties of the resulting hiPSC-ECs and whether hiPSC-ECs show different uptake than mature ECs. We discovered that all three hiPSC-EC lines showed similar nanoparticle uptake. On the other hand, we showed significantly lower uSPION uptake in hiPSC-ECs than in mature ECs. Data concerning differences between membrane properties of hiPSC-ECs and mature ECs have not been published. The most obvious interpretation suggests that one of the mechanisms responsible for mediation of endocytosis is not yet fully operational due to the immature state of the hiPSC-ECs. As we have previously confirmed by low density lipoprotein (LDL) assay, clathrin receptors are fully functional in hiPSC-ECs [13]. However, the state of caveolae receptors was not examined. We suspect that the immature state of the hiPSC-ECs could have significant effect on the expression of caveolae receptors. Whether the uptake will increase during maturation of the hiPSC-EC remains to be examined. 

uSPIONs were previously shown to be toxic [37,38]. Some studies showed dose dependent toxicity observed in concentration over 100 µg/mL, however others disagreed with these findings [39,40,41]. Because of this major toxicity concern we extensively studied the cytotoxicity of uSPIONs prepared in microwave plasma. We did not detect any acute effects of uSPIONs on the cell cultures. We did not show any cytotoxicity during the 6–48 h of exposure or any long-term toxic effects up to 12 days after incubation. We also tested long-term exposure to uSPIONs and did not detect any cytotoxicity. The lack of toxic effects can be explained by pre-treatment of uSPIONs in serum as serum pre-treatment was previously shown to significantly decrease uSPION reactivity, aggregation and cytotoxicity. Mahmoudi evaluated toxic effects of poly-vinylalcohol coated and uncoated nanoparticles in his work. He concluded that not coating, but rather protein corona is responsible for the observed toxic or lack of toxic effects [25,42]. It was shown repeatedly that protein corona plays a significant role in adsorption and endocytosis of SPIONs, while cell membrane does not come into contact directly with the nanoparticle surface but rather with the acquired protein corona. Therefore the presented results can be significantly influenced by incubation conditions during nanoparticle endocytosis or nanoparticle pre-treatment [29,30,31].

It was previously shown that SPIONs can affect cell properties. Chondrogenic and osteogenic differentiation of mesenchymal stem cells was reported to be affected, however different reports disagreed with these conclusions [43,44,45,46]. On the other hand, cardiogenic differentiation of hiPSCs was reported unaffected by SPIONs [47]. The exact mechanism that can interfere with the differentiation is unknown. Incubation with uSPIONs could influence hiPSC-ECs due to their immature state. Maintenance of phenotype and angiogenic properties are imperative for the use of endothelial cells. Therefore, we closely examined phenotype and function of hiPSC-ECs after the incubation with uSPIONs. We observed neither changes in cell phenotype nor angiogenic properties of the cells after the incubation with uSPIONs.

In conclusion, we studied labelling efficiency and cytotoxicity of uSPIONs prepared by microwave plasma. We showed efficient uSPION uptake and confirmed magnetic properties of endothelial cells even with low uSPION concentration. We did not observe any effect of uSPIONs on cell viability, phenotype or angiogenic properties of mature or hiPSC derived ECs. We observed lower uSPION uptake in hiPSC-ECs compared to mature ECs of the same genetic background. The difference can be explained by different membrane properties of hiPSC-ECs that can be attributed to their immature state. However, it remains to be examined whether maturation will resolve the issue. This work emphasizes the need of further studies to provide detailed characterization of hiPSCs-ECs. Despite the differences in uSPION uptake we conclude, that labelling hiPSC-ECs with low concentration of uSPIONs is feasible and does not show any toxic effects in vitro, which is an important step towards animal studies.

## 4. Materials and Methods

### 4.1. Cell Culture

All cell cultures were maintained in a 5% CO_2_ atmosphere at 37 °C. Cell cultures with 70–80% confluency were used for all listed experiments. 

HSVECs and HSVECs 1 were derived from patient samples (patients undergoing varicose vein surgery). The derived cells expressed endothelial markers (assessed by flow cytometry) and fulfilled the requirements of the endothelial functional assays—tube formation assay and LDL assay. HUVECs and HUVECs 1 (both from Thermo Fisher Scientific, Waltham, MA, USA) and HSVECs, HSVECs 1 were cultured in EGM2 (Lonza, Basel, Switzerland) according to the manufacturer’s instructions. hDFs (National Tissue Center Inc. Brno, Czech Republic), hDFs 1 (ThermoFischer Scientific, Waltham, MA, USA) were cultured in DMEM supplemented with 10% FBS, 100 µM non-essential amino acids, 1% penicillin/streptomycin, 2 mM l-glutamine and 0.1 mM mercaptoethanol (all components purchased from Life Technologies, Carlsbad, CA, USA). hiPSCs were cultured in colonies in mTeSRTM1 (Thermo Fisher Scientific) on MatrigelMT-coated dishes (Stemcell Technologies, Vancouver, Canada). All EC-lines differentiated from hiPSCs were cultivated in EGM2 (Lonza) supplemented with 50 ng/mL VEGF (PeproTech, Rocky Hill, NJ, USA). All cell cultures were routinely passaged at 80% confluency. 

### 4.2. Reprogramming and Differentiation of HUVECs, HSVECs and hDFs

hiPSCs were generated from HUVECs (hiPSCs-HU/CBIA-19), HUVECs 1 (hiPSCs-HU 1,/CBIA-29), HSVECs (hiPSCs-HS/CBIA-25), HSVECs (hiPSCs-HS 1/CBIA-37) and hDFs (hiPSCs-HF/CBIA-7), hDFs 1 (hiPSCs-HF 1/CBIA-15) using genome episomal vectors, based on the Epi5^TM^ Episomal hiPSC Reprogramming Kit (Life Technologies, Carlsbad, CA, USA) [48]. hiPSCs were maintained on Matrigel^TM^-precoated tissue-culture dishes in mTeSR^TM^1 medium. The medium was changed daily. All reprogrammed hiPSC lines expressed pluripotent markers, differentiated to all three germ layers and formed teratomas in immunodeficient mice [13,48,49].

Differentiation was performed according to previously published protocols [50]. The differentiated ECs (ECs-HU (1), ECs-HS (1), and ECs-HF (1)) expressed endothelial markers (CD31, CD144, KDR, and CD34), were able to take up acetylated low density lipoprotein (LDL) and maintained their angiogenic properties (tube formation assays were performed) [13]. For more information about reprogramming, differentiation and cultivation of hiPSC-ECs see Simara et al. [13].

### 4.3. Preparation and Analysis of uSPIONs

Maghemite nanoparticles were prepared from iron pentacarbonyle (Fe(CO)_5_), (99.99% purity, Sigma Aldrich, St. Louis, MO, USA) in a microwave plasma torch reactor [51,52]. Synthesis was performed in an argon gas flow; iron pentacarbonyl vapors were carried into the plasma reactor chamber and mixed with oxygen. In the reactor chamber, the mixture was introduced into microwave plasma, which reached high temperatures (up to 4000 K) and emitted strong UV radiation. As a result, the iron pentacarbonyl was fully decomposed to its atomic elements. Consequent reactions led to production of dry iron or iron oxide nanoparticles (depending on the stoichiometry of the oxygen and iron pentacarbonyl) and CO_2_. Particles were carefully collected from reactor walls after deposition a dry powder that contained nanoparticles of high crystallinity and chemical purity [52]. The production was tuned to specifically produce maghemite nanoparticles [52], and the operating parameters were 700 sccm for the argon gas flow, 1 sccm of iron pentacarbonyl and 50 sccm of oxygen with the reactor supplied by a 310 W microwave power.

Phase composition of prepared iron oxide samples was determined by X-ray powder diffraction (XRD) using a Rigaku X-ray diffractometer SmartLab Type F with a Cu cathode range of 20 to 120 2Ѳ degrees. XRD pattern was evaluated by PDXL2 software (Rigaku). As a supporting analysis to XRD, Raman spectroscopy was carried out using the HORIBA LabRAM HR Evolution system a with 532-m laser, using a 50× objective and 1% ND filter in the range from 120 to 1800 cm^−1^. The prepared nanopowder was imaged with Tescan scanning electron microscope MIRA3 with a Schottky field emission electron gun equipped with secondary electron (SE) and back-scattered electron (BSE) detectors as well as an Oxford Instruments EDX analyzer. 

The nanopowder consisted of cubic maghemite-C γ-Fe_2_O_3_ (ICSD #87119) with a small admixture of rhombohedral hematite α-Fe_2_O_3_ (ICSD #82137). The Rietveld refinement procedure (ending with Rwp = 3.24 and Ch^2 = 1.248) provided the following values for the aforementioned phases: the maghemite-C (a = 0.8355 nm, d_XRD_ = 22 nm, F = 96 wt%) and hematite α-Fe_2_O_3_ (a = 0.5035 nm, b = 0.5035 nm, c = 13. 752 nm, d_XRD_ = 20 nm, F = 4 wt%) phases. Raman spectra of the sample showed typical broad structures approximately 350, 500, 700 and 1350 cm^−1^ belonging to maghemite according Faria et al. [53] (Figure S1). Peaks at 223 and 293 cm^−1^ could be attributed to hematite in the sample. SEM analysis was carried out using a MIRA 3 microscope using a 15-kV accelerating voltage and SE detector. The nanopowder consisted of 20–50 nm uSPIONs and their aggregates, as shown in Figure S1. Polydispersity index (PDI) or dispersity was calculated as recommended by IUPAC, defined by ISO 22412:2017 for DLS and similar methods as

(1) $$\mathrm{PDI}={\left(\frac{\sigma }{D}\right)}^{2}$$

where D is average particle diameter and s its standard deviation. PDI was 0.18 which suggested moderate size distribution of maghemite nanoparticles.

The working stock of uSPIONs was prepared by resuspension of uSPIONs in FBS at a concentration of 1 mg/mL. The stock was incubated in an ultrasonic bath for 3 h to solubilize the nanoparticles properly. From the work stock, the uSPIONs were diluted to the desired concentration in cultivation medium. 

### 4.4. Prussian Blue Staining

Prussian blue staining is a histological test for iron. Six cell lines (HUVECs, HSVECs, hDFs, ECs-HU, ECs-HS, and ECs-NF) were incubated with three different concentrations of nanoparticles (1, 10, and 50 µg/mL). Samples were collected after 2, 4, 6, 24 and 48 h of incubation, fixed with 4% paraformaldehyde, stained with Prussian blue (Prussian Blue Reaction Kit, BioPAL, Worcester, MA, USA) and Red Nuclear Counterstain. They were inspected by light microscopy. 

### 4.5. MTT Assay

MTT assay is colorimetric assay based on the metabolism of the MTT substrate to a formazan crystal, an insoluble intracellular product that is measured after solubilization by spectrophotometry. Each cell type was seeded at a density of 10,000 cells per well in a 96-well plate and cultured for 24 h before exposure to the uSPIONs. Five different cell lines (HUVECs, HSVECs, ECs-HU, ECs-HS, and ECs-HF) were incubated with different concentrations of uSPIONs (10 and 50 µg/mL). Cell survival was assessed after 6, 24 and 48 h. For each cell line a sample cultured without uSPIONs was used as a control. At the end of the incubation period, new medium with MTT substrate was added into each well, and the MTT assays were performed according to the manufacturer’s specifications (Roche, Basel, Switzerland). 

### 4.6. Annexin Assay

Cell apoptosis was determined by Annexin assay. Cells were seeded 24 h prior to the start of the experiment. A total of 10 different cell lines (HUVECs (1), HSVECs (1), ECs-HU (1), ECs-HS (1), and ECs-HF(1)) were incubated with different nanoparticle concentrations (10 and 50 µg/mL), and cell survival was assessed after 6, 24 and 48 h. Annexin assays were performed according to the manufacturer’s instructions (Miltenyi, Bergisch Gladbach, Germany). 

### 4.7. Transmission Electron Microscopy 

HUVECs, HSVECs and hDFs were incubated with uSPIONs (10 µg/mL) or without uSPIONs (control) for 24 h, harvested using trypsin-EDTA and washed gently with PBS. To prepare samples for TEM, the cells were washed in 0.1 M cacodylate buffer, and fixed with 3% glutaraldehyde with 0.2% tantin in 0.1 M cacodylate buffer for 1 h. They were post-fixed with 1% OsO_4_, washed 3 times in 0.1 M cacodylate and embedded in 1% agar blocks. The blocks were dehydrated by a graded ethanol series (50%, 70%, 96% and 100%), treated with 100% acetone and embedded in Durcupan resin. Ultrathin sections were prepared on an LKB 8802A ultramicrotome, stained with OsO_4_ and examined with an FEI Morgagni 286(D) TEM.

### 4.8. Study of Magnetic Properties

The magnetic properties of HUVECs, HSVECs and ECs-HU were assessed by MACS separation (Miltenyi, Bergisch Gladbach, Germany). The separation was performed according to the manufacturer’s instructions. Cells were incubated with 10 µg/mL of uSPIONs for 24 h or 48 h, counted on a Beckman cell counter. They were separated according to their magnetic properties by MACS separation and counted on a Beckman counter again. Data are shown as ratio of the separated cells out of the population in percentage.

### 4.9. Flow-Cytometry Analysis of Endothelial Characteristics

Flow-cytometry analysis was performed on HUVECs, HSVECs and ECs-HU as follows. Cells were gathered, washed with PBS + EDTA (2 mM) + BSA (0.5%) and labeled in PBS + EDTA (2 mM) + BSA (0.5%) with CD31-APC, CD34-PE, CD144-PE and KDR-PE antibodies (Miltenyi, Bergisch Gladbach, Germany) for 10 min at 4 °C. The samples were analyzed by BD FACS Canto II flow cytometer (Becton–Dickinson, Heidelberg, Germany).

### 4.10. Image and Statistical Data Analysis

We evaluated the images in Matlab (MathWorks), and the automated routine analyzed the color images. Briefly, the pixels belonging to the cells were identified from the hue channel of the image converted to an HSV color model. First, we smoothed the hue channel with a Gaussian filter (sigma = 1). Then, we segmented the image using the multilevel Otsu thresholding method (considering three levels and taking the highest threshold). Finally, the components, which were bigger than 10 pixels of the binary mask, were morphologically closed with a structuring element with a radius of 11 pixels. The components larger than 1000 pixels were considered as the cell area.

The nuclear pixels were segmented in the a* channel (green-red) of the Lab color representation of the image. We applied the multilevel Otsu thresholding (3 levels, highest threshold) followed by morphological opening and closing with a structuring element with a radius of 11 pixels. Finally, we took the components that were larger than 1000 pixels. Particle pixels were identified as pixels having a red value for the RGB model that was lower than the blue value (decreased by 25, which was an empirically chosen parameter that worked well for all images).

We computed the number of uSPION pixels in the cytoplasmic regions (formed by cell pixels that were not also nuclear pixels) and normalized the cytoplasmic pixels to the number of cell nuclei to compute the number of pixels occupied by uSPIONs per cell. For statistical analysis, we used one-way ANOVA followed by Tukey’s test. We evaluated three independent biological replicates (one picture each) for each value; each picture contained at least 50 cells.

The Annexin and MTT assays were statistically analyzed by *t*-test, where each value representing three independent biological replicates was compared with the untreated control. 

### 4.11. Ethics Statement

Human tissue samples were collected under approved guidelines set by 1st Surgical Department, Faculty of Medicine, Masaryk University and St. Ann’s Hospital and by the National Tissue Centre, Brno. The project was approved by Multicenter Ethics Committee of Fakultní nemocnice u sv. Anny v Brně (St Anne University Hospital, Brno) works in compliance with its own rules of procedure and the ICH GCP. Its assessments of projects are generally based on the World Medical Association Declaration of Helsinki (WMA) and the International Ethical Guidelines for Biomedical Research Involving Human Subjects prepared by the Council for International Organizations of Medical Services (CIOMS) in cooperation with the World Health Organization (WHO). The reference number 8G/2016 date 13 April 2016. All patients signed an informed consent form. The authors proclaim that no tissue samples/organs were procured from prisoners.

## Supplementary Materials

Supplementary materials can be found at https://www.mdpi.com/1422-0067/20/14/3536/s1.

## Abbreviations

ECs	endothelial cells
EPCs	endothelial progenitor cells
SPIONs	superparamagnetic iron-oxide nanoparticles
uSPIONs	uncoated superparamagnetic iron-oxide nanoparticles
hiPSCs	human induced pluripotent stem cells
hiPSC-ECs	hiPSC-derived endothelial cells
HUVECs	human umbilical vein endothelial cells
hiPSC-HUs	hiPSC derived from human umbilical vein endothelial cells
ECs-HU	endothelial cells differentiated from hiPSC-HU
hDF	human dermal fibroblasts
hiPSC-HF	hiPSC derived from human dermal fibroblasts
ECs-HF	endothelial cells differentiated from hiPSC-HF
HSVECs	human saphena vein endothelial cells
hiPSC-HSs	hiPSC derived from human saphena vein endothelial cells
ECs-HS	endothelial cells differentiated from hiPSC-HSs
ROS	reactive oxygen species
TEM	transmission electron microscopy

## References

[1] Chong M.S.K., Ng W.K., Chan J.K.Y. (2016). Concise Review: Endothelial Progenitor Cells in Regenerative Medicine: Applications and Challenges. Stem Cells Transl. Med..

[2] Asahara T., Kawamoto A., Masuda H. (2011). Concise Review: Circulating Endothelial Progenitor Cells for Vascular Medicine. Stem Cells.

[3] Friedrich E.B., Walenta K., Scharlau J., Nickenig G., Werner N. (2006). CD34-/CD133+/VEGFR-2+ endothelial progenitor cell subpopulation with potent vasoregenerative capacities. Circ. Res..

[4] Hirata K., Li T.S., Nishida M., Ito H., Matsuzaki M., Kasaoka S., Hamano K. (2003). Autologous bone marrow cell implantation as therapeutic angiogenesis for ischemic hindlimb in diabetic rat model. Am. J. Physiol. Heart Circ. Physiol..

[5] Polyak B., Fishbein I., Chorny M., Alferiev I., Williams D., Yellen B., Friedman G., Levy R.J. (2008). High field gradient targeting of magnetic nanoparticle-loaded endothelial cells to the surfaces of steel stents. Proc. Natl. Acad. Sci. USA.

[6] Fadini G.P., Miorin M., Facco M., Bonamico S., Baesso I., Grego F., Menegolo M., de Kreutzenberg S.V., Tiengo A., Agostini C. (2005). Circulating Endothelial Progenitor Cells Are Reduced in Peripheral Vascular Complications of Type 2 Diabetes Mellitus. J. Am. Coll. Cardiol..

[7] Peichev M., Naiyer A.J., Pereira D., Zhu Z., Lane W.J., Williams M., Oz M.C., Hicklin D.J., Witte L., Moore M.A. (2000). Expression of VEGFR-2 and AC133 by circulating human CD34(+) cells identifies a population of functional endothelial precursors. Blood.

[8] Hibino N., Duncan D.R., Nalbandian A., Yi T., Qyang Y., Shinoka T., Breuer C.K. (2012). Evaluation of the use of an induced puripotent stem cell sheet for the construction of tissue-engineered vascular grafts. J. Thorac. Cardiovasc. Surg..

[9] Simara P., Motl J.A., Kaufman D.S. (2013). Pluripotent stem cells and gene therapy. Transl. Res..

[10] Nakayama K.H., Joshi P.A., Lai E.S., Gujar P., Joubert L.M., Chen B., Huang N.F. (2015). Bilayered vascular graft derived from human induced pluripotent stem cells with biomimetic structure and function. Regen. Med..

[11] Bao X., Lian X., Dunn K.K., Shi M., Han T., Qian T., Bhute V.J., Canfield S.G., Palecek S.P. (2015). Chemically-defined albumin-free differentiation of human pluripotent stem cells to endothelial progenitor cells. Stem Cell Res..

[12] Prasain N., Lee M.R., Vemula S., Meador J.L., Yoshimoto M., Ferkowicz M.J., Fett A., Gupta M., Rapp B.M., Saadatzadeh M.R. (2014). Differentiation of human pluripotent stem cells to cells similar to cord-blood endothelial colony-forming cells. Nat. Biotechnol..

[13] Simara P., Tesarova L., Rehakova D., Farkas S., Salingova B., Kutalkova K., Vavreckova E., Matula P., Matula P., Veverkova L. (2017). Reprogramming of adult peripheral blood cells into human induced pluripotent stem cells as a safe and accessible source of endothelial cells. Stem Cells Dev..

[14] Orlova V.V., Drabsch Y., Freund C., Petrus-Reurer S., van den Hil F.E., Muenthaisong S., Dijke P.T., Mummery C.L. (2014). Functionality of endothelial cells and pericytes from human pluripotent stem cells demonstrated in cultured vascular plexus and zebrafish xenografts. Arter. Thromb. Vasc. Biol..

[15] Samuel R., Daheron L., Liao S., Vardam T., Kamoun W.S., Batista A., Buecker C., Schäfer R., Han X., Au P. (2013). Generation of functionally competent and durable engineered blood vessels from human induced pluripotent stem cells. Proc. Natl. Acad. Sci. USA.

[16] Ali A., Zafar H., Zia M., Ul Haq I., Phull A.R., Ali J.S., Hussain A. (2016). Synthesis, characterization, applications, and challenges of iron oxide nanoparticles. Nanotechnol. Sci. Appl..

[17] Anselmo A.C., Mitragotri S. (2016). Nanoparticles in the clinic. Bioeng. Transl. Med..

[18] Chen R., Yu H., Jia Z.Y., Yao Q.L., Teng G.J. (2011). Efficient nano iron particle-labeling and noninvasive MR imaging of mouse bone marrow-derived endothelial progenitor cells. Int. J. Nanomed..

[19] Soenen S.J., Himmelreich U., Nuytten N., De Cuyper M. (2011). Cytotoxic effects of iron oxide nanoparticles and implications for safety in cell labelling. Biomaterials.

[20] Castaneda R.T., Khurana A., Khan R., Daldrup-Link H.E. (2011). Labeling stem cells with ferumoxytol, an FDA-approved iron oxide nanoparticle. J. Vis. Exp. Jove.

[21] Bogart L.K., Pourroy G., Murphy C.J., Puntes V., Pellegrino T., Rosenblum D., Peer D., Lévy R. (2014). Nanoparticles for imaging, sensing, and therapeutic intervention. ACS Nano.

[22] Rejman J., Oberle V., Zuhorn I.S., Hoekstra D. (2004). Size-dependent internalization of particles via the pathways of clathrin- and caveolae-mediated endocytosis. Biochem. J..

[23] Calero M., Gutiérrez L., Salas G., Luengo Y., Lázaro A., Acedo P., Morales M.P., Miranda R., Villanueva A. (2014). Efficient and safe internalization of magnetic iron oxide nanoparticles: two fundamental requirements for biomedical applications. Nanomedicine.

[24] Kettler K., Veltman K., van de Meent D., van Wezel A., Hendriks A.J. (2014). Cellular uptake of nanoparticles as determined by particle properties, experimental conditions, and cell type. Environ. Toxicol. Chem..

[25] Mahmoudi M., Simchi A., Imani M., Shokrgozar M.A., Milani A.S., Häfeli U.O., Stroeve P. (2010). A new approach for the in vitro identification of the cytotoxicity of superparamagnetic iron oxide nanoparticles. Colloids Surf. B Biointerfaces.

[26] Bashir M.R., Bhatti L., Marin D., Nelson R.C. (2015). Emerging applications for ferumoxytol as a contrast agent in MRI. J. Magn. Reson. Imaging.

[27] Shahnaz G., Kremser C., Reinisch A., Vetter A., Laffleur F., Rahmat D., Iqbal J., Dünnhaupt S., Salvenmoser W., Tessadri R. (2013). Efficient MRI labeling of endothelial progenitor cells: design of thiolated surface stabilized superparamagnetic iron oxide nanoparticles. Eur. J. Pharm. Biopharm..

[28] Wei M.Q., Wen D.D., Wang X.Y., Huan Y., Yang Y., Xu J., Cheng K., Zheng M.W. (2015). Experimental study of endothelial progenitor cells labeled with superparamagnetic iron oxide in vitro. Mol. Med. Rep..

[29] Nguyen V.H., Lee B.-J. (2017). Protein corona: A new approach for nanomedicine design. Int. J. Nanomed..

[30] Carril M., Padro D., del Pino P., Carrillo-Carrion C., Gallego M., Parak W.J. (2017). In situ detection of the protein corona in complex environments. Nat. Commun..

[31] Wang F., Yu L., Monopoli M.P., Sandin P., Mahon E., Salvati A., Dawson K.A. (2013). The biomolecular corona is retained during nanoparticle uptake and protects the cells from the damage induced by cationic nanoparticles until degraded in the lysosomes. Nanomed. Nanotechnol. Biol. Med..

[32] Conner S.D., Schmid S.L. (2003). Regulated portals of entry into the cell. Nature.

[33] Behzadi S., Serpooshan V., Tao W., Hamaly M.A., Alkawareek M.Y., Dreaden E.C., Brown D., Alkilany A.M., Farokhzad O.C., Mahmoudi M. (2017). Cellular uptake of nanoparticles: Journey inside the cell. Chem. Soc. Rev..

[34] Zhang S., Li J., Lykotrafitis G., Bao G., Suresh S. (2009). Size-Dependent Endocytosis of Nanoparticles. Adv Mater..

[35] Roth T.F., Porter K.R. (1964). Yolk protein uptake in the oocyte of the mosquito *Aedes aegypti.* L.. J. Cell Biol..

[36] Zhang S., Gao H., Bao G. (2015). Physical Principles of Nanoparticle Cellular Endocytosis. ACS Nano.

[37] Hanini A., Schmitt A., Kacem K., Chau F., Ammar S., Gavard J. (2011). Evaluation of iron oxide nanoparticle biocompatibility. Int. J. Nanomed..

[38] Singh N., Jenkins G.J., Asadi R., Doak S.H. (2010). Potential toxicity of superparamagnetic iron oxide nanoparticles (SPION). Nano Rev..

[39] Buyukhatipoglu K., Clyne A.M. (2011). Superparamagnetic iron oxide nanoparticles change endothelial cell morphology and mechanics via reactive oxygen species formation. J. Biomed. Mater. Res. A.

[40] Pongrac I.M., Pavičić I., Milić M., Brkić Ahmed L., Babič M., Horák D., Vinković Vrček I., Gajović S. (2016). Oxidative stress response in neural stem cells exposed to different superparamagnetic iron oxide nanoparticles. Int. J. Nanomed..

[41] Zhu X.M., Wang Y.X., Leung K.C., Lee S.F., Zhao F., Wang D.W., Lai J.M., Wan C., Cheng C.H., Ahuja A.T. (2012). Enhanced cellular uptake of aminosilane-coated superparamagnetic iron oxide nanoparticles in mammalian cell lines. Int. J. Nanomed..

[42] Clift M.J.D., Bhattacharjee S., Brown D.M., Stone V. (2010). The effects of serum on the toxicity of manufactured nanoparticles. Toxicol. Lett..

[43] Arbab A.S., Yocum G.T., Rad A.M., Khakoo A.Y., Fellowes V., Read E.J., Frank J.A. (2005). Labeling of cells with ferumoxides-protamine sulfate complexes does not inhibit function or differentiation capacity of hematopoietic or mesenchymal stem cells. NMR Biomed..

[44] Kostura L., Kraitchman D.L., Mackay A.M., Pittenger M.F., Bulte J.W. (2004). Feridex labeling of mesenchymal stem cells inhibits chondrogenesis but not adipogenesis or osteogenesis. NMR Biomed..

[45] Zeng G., Wang G., Guan F., Chang K., Jiao H., Gao W., Xi S., Yang B. (2011). Human amniotic membrane-derived mesenchymal stem cells labeled with superparamagnetic iron oxide nanoparticles: The effect on neuron-like differentiation in vitro. Mol. Cell. Biochem..

[46] Chen Y.C., Hsiao J.K., Liu H.M., Lai I.Y., Yao M., Hsu S.C., Ko B.S., Yang C.S., Huang D.M. (2010). The inhibitory effect of superparamagnetic iron oxide nanoparticle (Ferucarbotran) on osteogenic differentiation and its signaling mechanism in human mesenchymal stem cells. Toxicol. Appl. Pharm..

[47] Au K.W., Liao S.Y., Lee Y.K., Lai W.H., Ng K.M., Chan Y.C., Yip M.C., Ho C.Y., Wu E.X., Li R.A. (2009). Effects of iron oxide nanoparticles on cardiac differentiation of embryonic stem cells. Biochem. Biophys. Res. Commun..

[48] Šimara P., Tesařová L., Padourová S., Koutná I. (2014). Generation of human induced pluripotent stem cells using genome integrating or non-integrating methods. Folia Biol. Praha.

[49] Simara P., Tesarova L., Rehakova D., Matula P., Stejskal S., Hampl A., Koutna I. (2017). DNA double-strand breaks in human induced pluripotent stem cell reprogramming and long-term in vitro culturing. Stem Cell Res..

[50] Orlova V.V., van den Hil F.E., Petrus-Reurer S., Drabsch Y., Ten Dijke P., Mummery C.L. (2014). Generation, expansion and functional analysis of endothelial cells and pericytes derived from human pluripotent stem cells. Nat. Protoc..

[51] Synek P., Jasek O., Zajickova L. (2014). Study of Microwave Torch Plasmachemical Synthesis of Iron Oxide Nanoparticles Focused on the Analysis of Phase Composition. Plasma Chem. Plasma Process..

[52] Synek P., Jasek O., Zajickova L., Kudrle V., Pizurova N. (2011). Plasmachemical synthesis of maghemite nanoparticles in atmospheric pressure microwave torch. Mater. Lett..

[53] de Faria D.L.A., Venâncio S.S., de Oliveira M.T. (1997). Raman microspectroscopy of some iron oxides and oxyhydroxides. J. Rom. Spectrosc..

