# Supplementary material for: The Effect of Uncoated SPIONs on hiPSC-Differentiated Endothelial Cells
Source: Int J Mol Sci. 2019 Jul 19;20(14):3536. doi: 10.3390/ijms20143536 (PMC6678752; doi:10.3390/ijms20143536)
Supplement: Supplementary file 1 [file ijms-20-03536-s001.pdf]

Supplementary material

The effect of uncoated SPIONs on hiPSC-differentiated endothelial cells

*Barbara Salingova[1], Pavel Simara[1], Pavel Matula[1], Lenka Zajickova[2], Petr Synek[3], Ondrej Jasek[3], Lenka Veverkova[4], Miroslava Sedlackova[5], Zuzana Nichtova[6], Irena Koutna[1]**

[1] Centre for Biomedical Image Analysis, Faculty of Informatics, Masaryk University, Kamenice 5, 625 00, Czech Republic
[2] RG Plasma Technologies, CEITEC – Masaryk University, Purkynova 656/123, 612 00, Czech Republic
[3] Department of Physical Electronics, Faculty of Science, Masaryk University, (Kotlarska 2, Brno 602 00, Czech Republic
[4] 1st Surgical Department, Faculty of Medicine, Masaryk University and St. Ann's Hospital, Pekarska 53, 656 91 Brno, Czech Republic
[5] Department of Histology and Embryology, Faculty of Medicine, Masaryk University, Kamenice 5, 625 00 Brno, Czech Republic
[6] Institute of Molecular Physiology and Genetics, Slovak Academy of Sciences, Dubravska cesta 9, 840 05 Bratislava, Slovak Republic
*Correspondence: Assoc. prof. Irena Koutna PhD., qkoutna@gmail.com (+420) 549 493 976

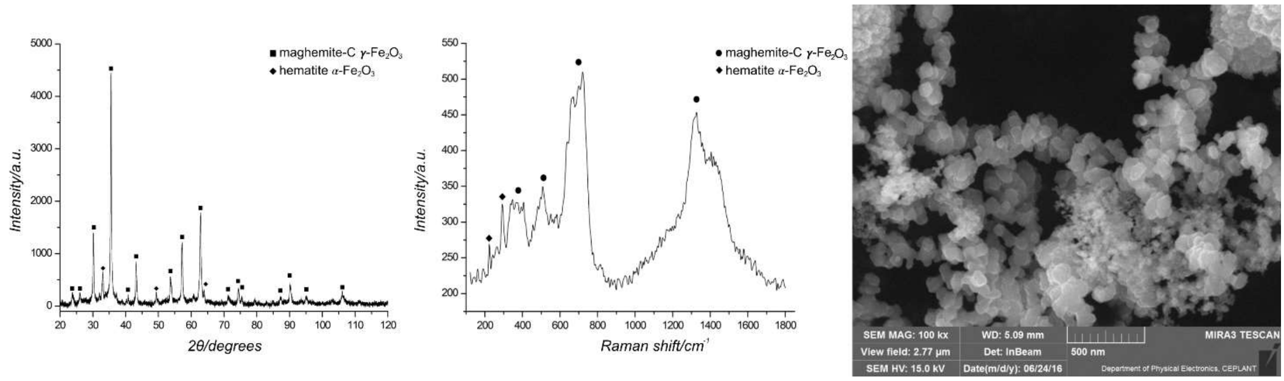

Figure S1 *Characterisation of the prepared uSPIONs. Left, sample diffraction pattern. Middle, phase composition of the prepared sample analyzed by Raman spectroscopy. Right, SEM of the synthetized uSPIONs (size: 20-50 nm). Abbreviations: SEM, scanning electron microscopy.*

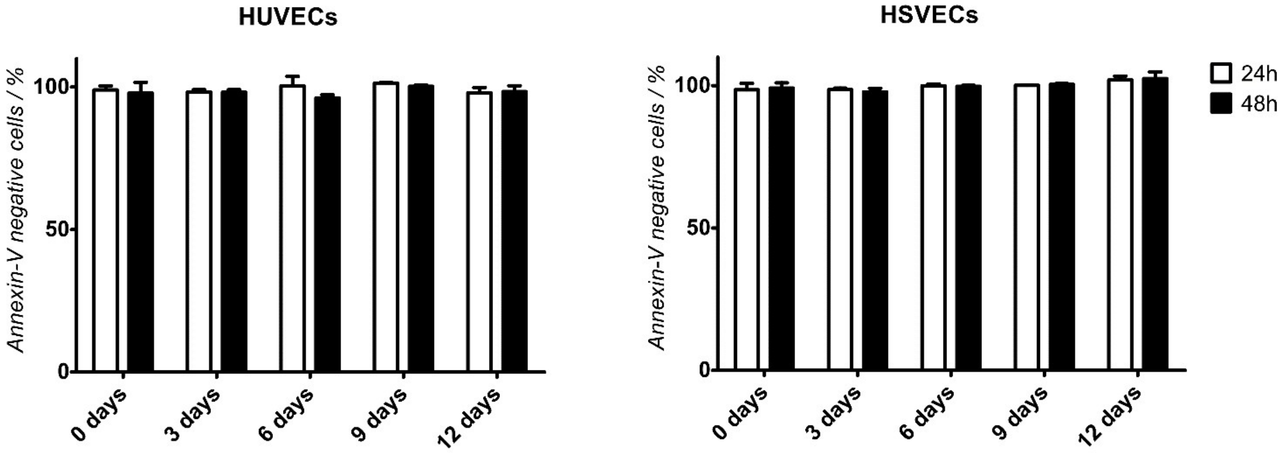

Figure S2 *Cell viability after incubation with uSPIONs assessed by Annexin assay. HSVECs and HUVECs were incubated with 10 µg/ml uSPIONs for 24 and 48 h and observed up to 12 days after incubation. Viability was assessed by annexin assay during passaging – every 3 days (N= ±3 SEM). Viability was standardized to the control. The statistical evaluation (t-test) showed no significant difference in cell survival among the cell lines (p> 0.05). Abbreviations: HUVECs, human umbilical vein endothelial cells; HSVECs, human saphenous vein endothelial cells.*

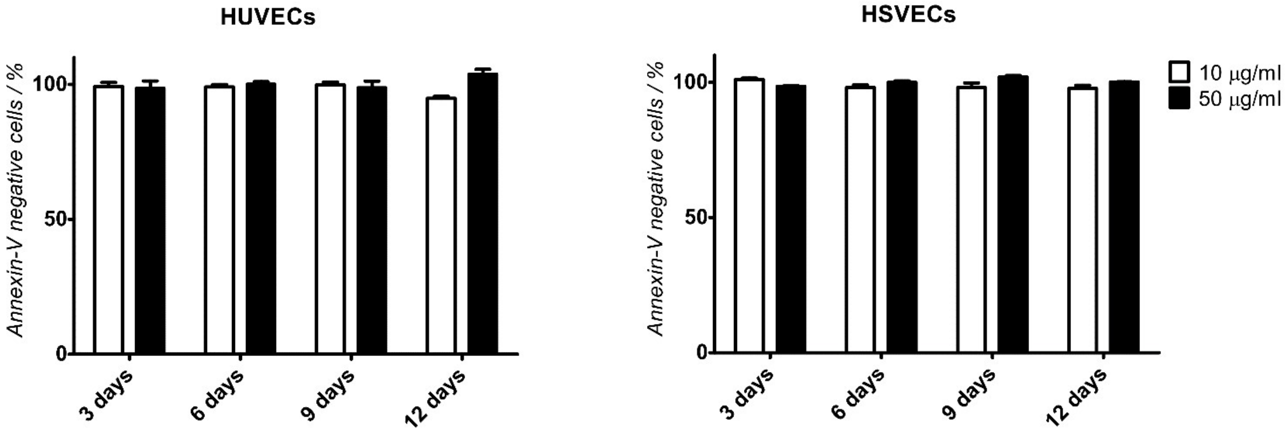

Figure S3 *Cell viability during long-term incubation with uSPIONs assessed by Annexin assay. HUVECs and HSVECs were incubated with 10 and 50 µg/ml uSPIONs for 3, 6, 9 and 12 days (N= ±3 SEM). Viability was standardized to the control. The statistical evaluation (t-test) showed no significant difference in cell viability among the cell lines (p> 0.05). Abbreviations: HUVECs, human umbilical vein endothelial cells; HSVECs, human saphenous vein endothelial cells.*

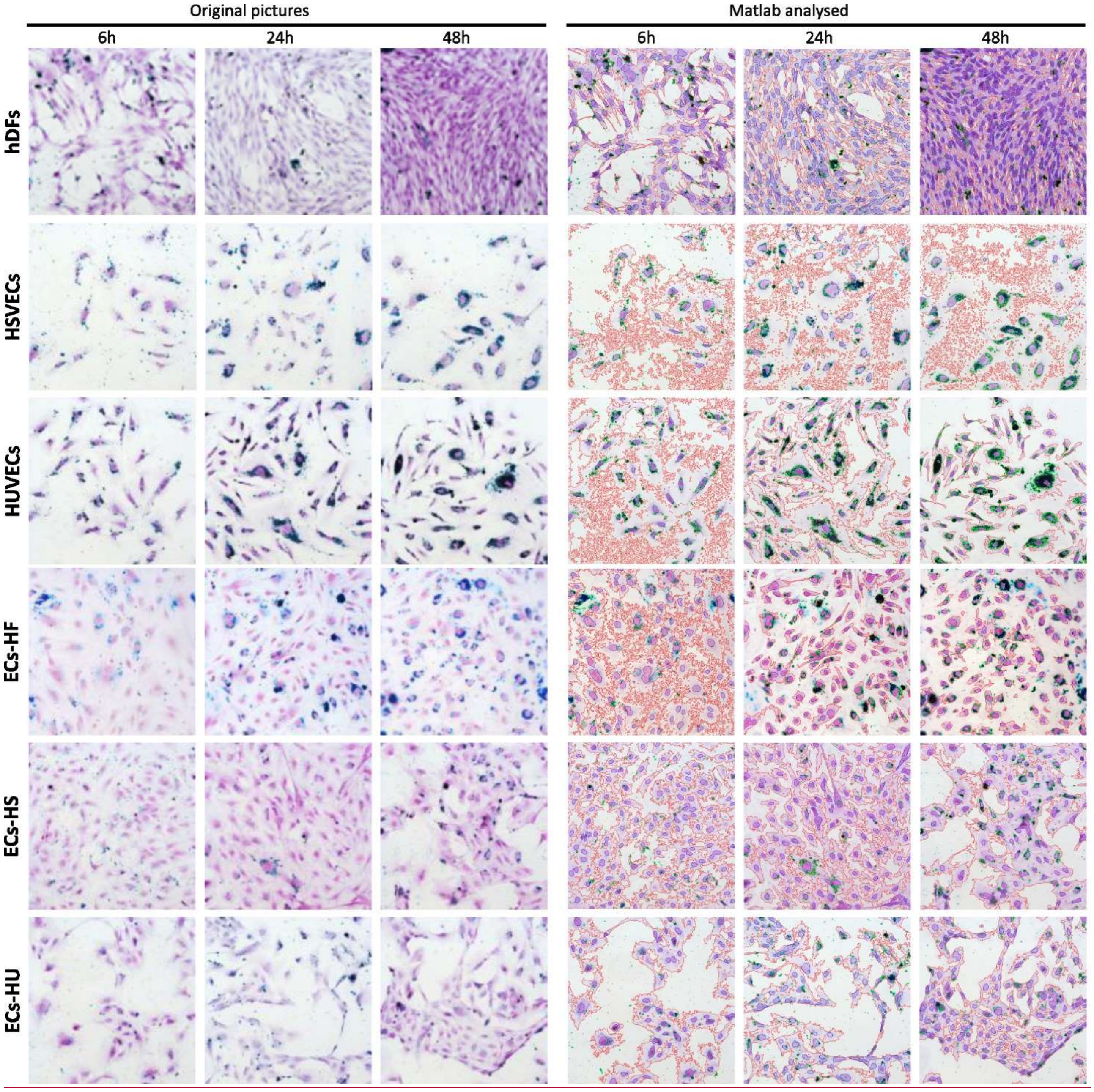

Figure S4 Internalization of uSPIONs in 6 cell types, Matlab analysis. *Left - Representative pictures of internalization of uSPIONs visualized after Prussian blue staining. Cells were incubated with uSPIONs for 6 h, 24 h and 48 h, stained with Prussian blue for iron detection and observed under a light microscope. Right – Matlab analysis of internalization of uSPIONs visualized after Prussian blue staining. Analysis of number of uSPIONs inside the cell. Red lines – cell boarders, green – uSPIONs. Abbreviations: HUVECs, human umbilical vein endothelial cells; HSVECs, human saphenous vein endothelial cells; hDFs, adult human dermal fibroblasts; ECs-HUs, endothelial cells differentiated from hiPSCs-HU; ECs-HS, endothelial cells differentiated from hiPSCs-HS; ECs-HF, endothelial cells differentiated from hiPSCs-HF.*
